# The use of dedicated long-axis views focused on the left atrium improves the accuracy of left atrial volumes and emptying fraction measured by cardiovascular magnetic resonance

**DOI:** 10.1186/s12968-022-00905-w

**Published:** 2023-02-16

**Authors:** Lara Tondi, Luigi P. Badano, Stefano Figliozzi, Silvia Pica, Camilla Torlasco, Antonia Camporeale, Diana R. Florescu, Giandomenico Disabato, Gianfranco Parati, Massimo Lombardi, Denisa Muraru

**Affiliations:** 1 Multimodality Cardiac Imaging Section, IRCCS Policlinico San Donato, San Donato Milanese, Milan, Italy; 2 Department of Cardiology, Istituto Auxologico Italiano IRCCS, Milan, Italy; 3 Department of Medicine and Surgery, University Milano-Bicocca, Milan, Italy; 4 Clinical Echocardiography Diagnostic Service, Cardio Center, Humanitas Research Hospital IRCCS, Rozzano, Milan Italy; 5 Department of Cardiology, University of Medicine and Pharmacy of Craiova, Craiova, Romania; 6 University of Pavia, Pavia, Italy

**Keywords:** Left atrial volume, Left atrial emptying fraction, Left atrial strain, Cardiac magnetic resonance, Accuracy

## Abstract

**Background:**

The use of apical views focused on the left atrium (LA) has improved the accuracy of LA volume evaluation by two-dimensional (2D) echocardiography. However, routine cardiovascular magnetic resonance (CMR) evaluation of LA volumes still uses standard 2- and 4-chamber cine images focused on the left ventricle (LV). To investigate the potential of LA-focused CMR cine images, we compared LA maximuml (LAVmax) and minimum (LAVmin) volumes, and emptying fraction (LAEF), calculated on both standard and LA-focused long-axis cine images, with LA volumes and LAEF obtained by short-axis cine stacks covering the LA. LA strain was also calculated and compared between standard and LA-focused images.

**Methods:**

LA volumes and LAEF were obtained from 108 consecutive patients by applying the biplane area-length algorithm to both standard and LA-focused 2- and 4-chamber cine images. Manual segmentation of a short-axis cine stack covering the LA was used as the reference method. In addition, LA strain reservoir (εs), conduit (εe) and booster pump (εa) were calculated using CMR feature-tracking**.**

**Results:**

Compared to the reference method, the standard approach significantly underestimated LA volumes (LAVmax: bias − 13 ml; LOA =  + 11, − 37 ml; LAVmax i: bias − 7 ml/m^2^; LOA =  + 7, − 21 ml/m^2^; LAVmin; bias − 10 ml, LOA: + 9, − 28 ml; LAVmin i: bias − 5 ml/m^2^, LOA: + 5, − 16 ml/m^2^), and overestimated LA-EF (bias 5%, LOA: + 23, − 14%). Conversely, LA volumes (LAVmax: bias 0 ml; LOA: + 10, − 10 ml; LAVmax i: bias 0 ml/m^2^; LOA: + 5, − 6 ml/m^2^; LAVmin: bias − 2 ml; LOA: + 7, − 10 ml; LAVmin i: bias − 1 ml/m^2^; LOA: + 3, − 5 ml/m^2^) and LAEF (bias 2%, LOA: + 11, − 7%) by LA-focused cine images were similar to those measured using the reference method. LA volumes by LA-focused images were obtained faster than using the reference method (1.2 vs 4.5 min, p < 0.001). LA strain (εs: bias 7%, LOA = 25, − 11%; εe: bias 4%, LOA = 15, − 8%; εa: bias 3%, LOA = 14, − 8%) was significantly higher in standard vs. LA-focused images (p < 0.001).

**Conclusion:**

LA volumes and LAEF measured using dedicated LA-focused long-axis cine images are more accurate than using standard LV-focused cine images. Moreover, LA strain is significantly lower in LA-focused vs. standard images.

**Supplementary Information:**

The online version contains supplementary material available at 10.1186/s12968-022-00905-w.

## Background

Left atrial (LA) size and function are robust prognostic markers of adverse cardiovascular outcomes in a variety of cardiac conditions [1–6].

Cardiovascular magnetic resonance (CMR) is considered the reference imaging modality to assess the size and function of cardiac chambers [7], and recently LA size and function measured by CMR have been reported to be associated with cardiovascular outcome, independent of left ventricular (LV) measures [5]. However, current CMR guidelines do not provide specific recommendations on how to measure LA volumes [8]. LA cavity contouring from a short-axis cine stack has been reported to provide larger LA volumes than the conventional biplane area-length algorithm applied to standard (i.e. images focused on the LV) long-axis 2- and 4-chamber cine images [9]. Short axis contouring  has been used as a reference method to validate LA volumes and emptying fraction (LAEF) measured by three-dimensional (3D) echocardiography [10], and to assess the prognostic value of LA function in survivors of a ST elevation myocardial infarction [11]. However, the short axis contouring method has never been implemented in routine CMR practice, due to its significant scan acquisition and post-processing time. As a result, LA volumes and strain by CMR are usually evaluated by applying the biplane area-length algorithm and the feature tracking method to standard long-axis 2- and 4-chamber cine images [1, 2, 7, 12–14]. However, the latter are oriented according to the major axis of the LV, which lies in a plane with a different spatial orientation compared to the LA [10]. Thus, the standard long-axis views optimized according to the maximal LV length commonly foreshorten the LA, with the consequent underestimation its size and overestimation of the extent of myocardial wall deformation by strain. [10, 15–17]

A previous transthoracic echocardiographic study showed that apical 2- and 4-chamber views focused on the LA provided more accurate estimation of LA maximum volume (LAVmax) than standard long-axis views [16]. However, it remains to be clarified whether the acquisition of dedicated LA-focused long-axis cine views improves the accuracy of LA volumes estimation, and the impact of these additional acquisitions on the current workflow in CMR daily practice. To address these issues, we designed a prospective, observational, multicenter study to: (i) assess whether the use of LA-focused long-axis images improves the accuracy in quantifying LA volumes and LAEF, compared to standard long-axis images; (ii) quantify both the acquisition time required to obtain LA focused long-axis images and the computational time to obtain LA volumes and LAEF (iii) and compare LA strain values obtained by standard vs. LA-focused long-axis images.

## Methods

### Study design

The present investigation is a prospective, multicenter and observational study. Consecutive patients undergoing clinically referred CMR at Istituto Auxologico Italiano, IRCSS (Milan, Italy), and Policlinico San Donato, IRCSS (San Donato Milanese, Italy), were screened for study inclusion from May 2021 to June 2021. Exclusion criteria were: (i) age < 18 years; (ii) significant cardiac arrhythmia (i.e. atrial fibrillation); (iii) unwillingness to take part in the study; (iv) congenital heart disease; (v) pregnancy and/or any other contraindication to CMR study; (vi) heart transplantation; (vii) non-diagnostic image quality. Demographics and clinical data were recorded on the same day of CMR scan.

The study was performed according to the principles of the declaration of Helsinki and was approved by the Ethics Committee of the Istituto Auxologico Italiano, IRCCS, Milan, Italy (reference # CE 2021_05_18_09, approved on May 18th, 2021). All patients gave written consent to have their anonymized clinical data used for scientific purposes. The data that support the findings of this study are available on reasonable request to the corresponding author.

### CMR study protocol

CMR were performed on 1.5 T system (MAGNETOM Aera at Policlinico San Donato, and AVANTOFit at Istituto Auxologico Italiano; Siemens Healthineers, Erlangen, Germany). LV volumes, mass (LVM) and ejection fraction (LVEF), and right ventricular (RV) volumes and ejection fraction (RVEF) were obtained from manual tracing of cine balanced steady-state free precession (bSSFP) images acquired at the end of expiration, using the following parameters: slice thickness 6.0 mm, no gap, flip angle 60°–80°, repetition time and echo time were tailored for each patient to achieve 25 to 30 cardiac phases per cardiac cycle; typical readout field of view = 350 mm; phase resolution matrix = 75%; voxel size = 1.4 × 1.4 mm.

#### CMR acquisition of the LA

**Fig. 1:**
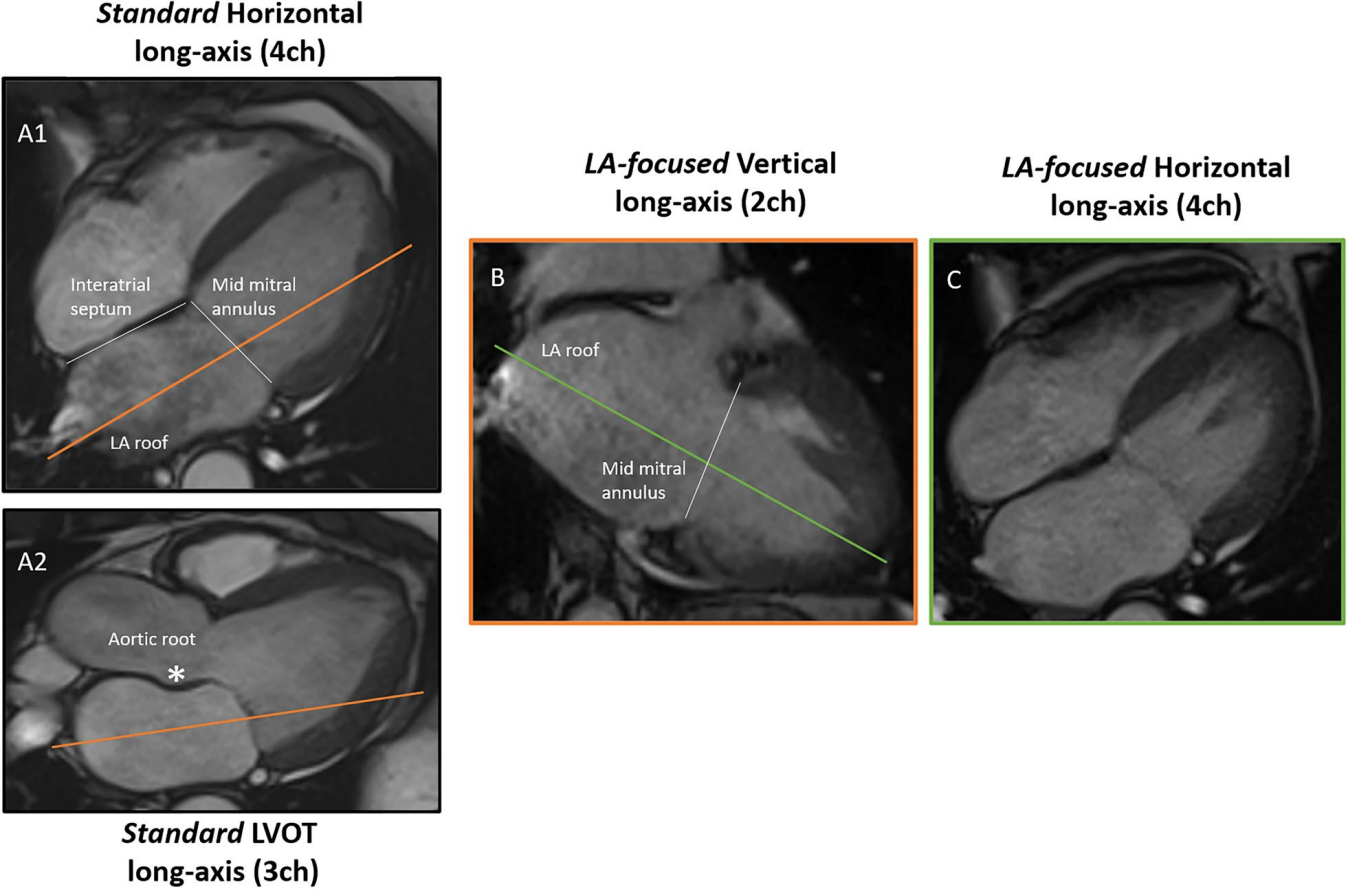
Method used to obtain the left-atrial focused long-axis cine images. Left atrial (LA)-focused 4- and 2-chamber long-axis cine images were planned from the standard long-axis cine images. Starting from the end-systolic standard 4-chamber cine image (**A1**), the LA-focused 2-chamber image (**B**) was obtained, oriented parallel to the interatrial septum, by selecting a perpendicular plane passing through the roof of the LA and the center of the mitral annulus. From the cine image in panel B, a perpendicular plane was selected, again intercepting the roof of the LA and the center of the mitral annulus, to obtain the LA-focused 4-chamber cine image (**C**). Before acquisition, the plane orientation to obtain LA-focused cine images was cross-checked in the standard 3-chamber cine image (**A2**), to avoid the inclusion of the adjacent aortic root in the final images

Cine bSSFP images of the LA were obtained using 3 protocols:

- 1. The standard (i.e. LV-focused) 2-chamber long-axis views were obtained from the vertical long-axis scout already acquired with modification to pass through the anterior and inferior LV myocardial walls [8]. The standard 4-chamber long-axis cine images were obtained from the 2-chamber long-axis views, through the LV apex and the center of the mitral annulus.
- 2. The LA-focused 4- and 2-chamber long-axis cine images were planned from the standard long-axis cine images. The method used to produce LA-focused cine images is explained in Fig. 1. By starting from the end-systolic standard 4-chamber cine image (Fig. 1, panel A1), the LA-focused 2-chamber image (Fig. 1, panel B) was obtained, oriented parallel to the interatrial septum, by selecting a perpendicular plane passing through the roof of the LA and the center of the mitral annulus. From the cine image in panel B, a perpendicular plane was selected, again intercepting the roof of the LA and the center of the mitral annulus, to obtain the LA-focused 4-chamber cine image (Fig. 1, panel C). Before the acquisition, the plane orientation to obtain LA- focused cine images was cross-checked in the standard 3-chamber cine image (Fig. 1, Panel A2), to avoid the inclusion of the adjacent aortic root in the final images.
- 3. The reference used for LA volumes and LAEF was a short-axis stack of cine images covering the entire LA, from the atrio-ventricular ring to the LA base. Orientation of the short-axis stack was perpendicular to the interatrial septum. Slice thickness was 6 mm with no gap and with temporal resolution < 45 ms [8, 18].

**Fig. 2:**
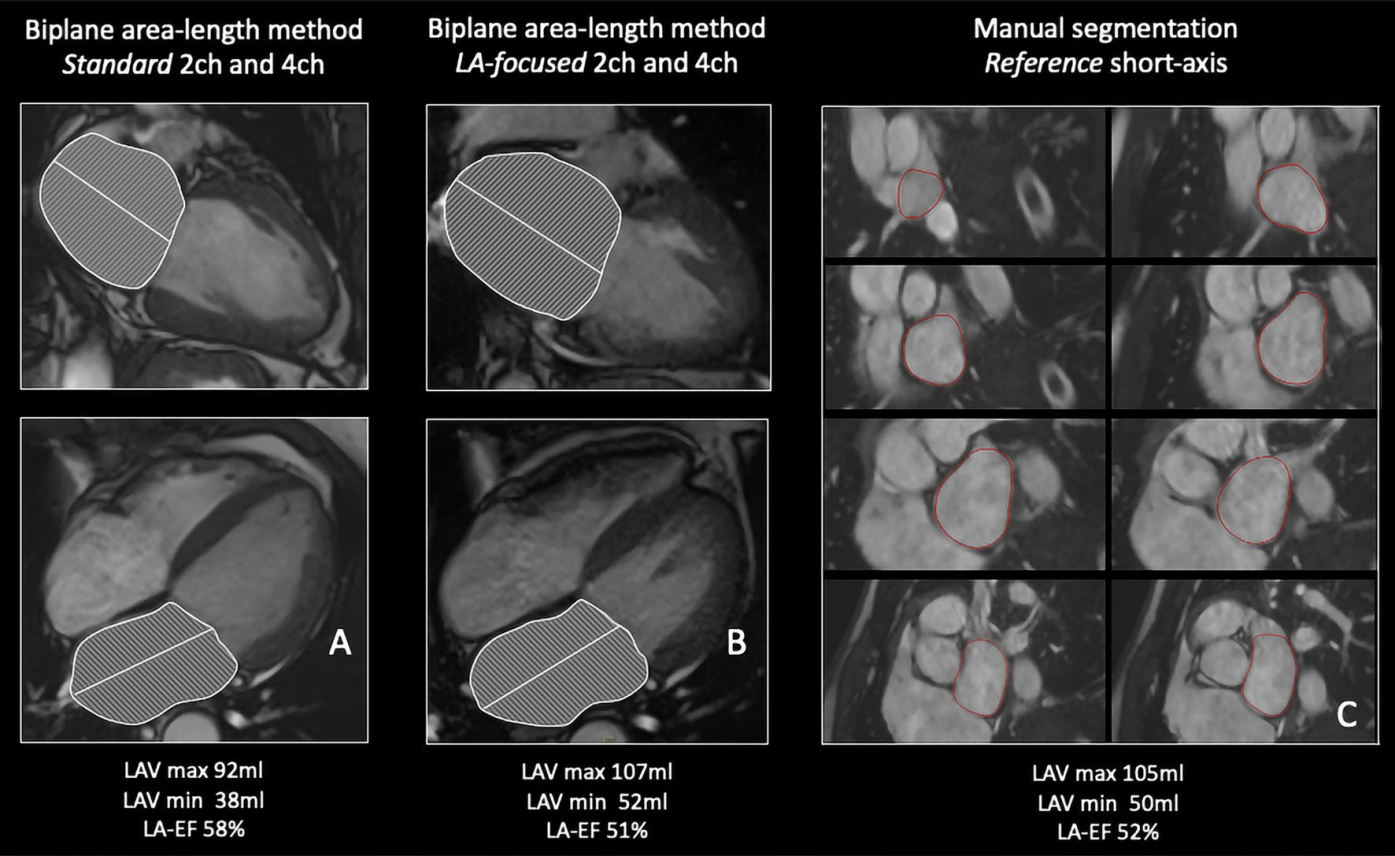
LA volumes and left atrial emptying fraction (LAEF) quantification on standard (**A**), left-atrial focused (**B**) and reference short-axis images (**C**). The biplane area-length method was applied to **A** and **B** while **C** was analyzed using the Simpson’s disc summation algorithm. All images represent end-systolic frames. In the standard images (**A**) the LA is clearly foreshortened compared to the LA focused cine images (**B**).  LAVmin: Minimum left atrial volume; LAVMax: Maximimum left atrial volume

An example of reference, standard and LA-focused cine images is illustrated in Fig. 2.

The time delays of the CMR scan due to LA-focused long-axis cine images and LA short-axis cine stack acquisitions were measured.

#### CMR post-processing of the LA

Analysis of the LA included LAVmax, minimum LA volume (LAVmin), LAEF and LA strain. LA volumes were reported both as absolute values and as indexed to body surface area.

In both standard and LA-focused long-axis cine-images, the LA cavity borders were traced in 4- and 2-chamber long-axis views in the frame before the opening of the mitral valve (LA end-diastole), and in the frame corresponding to the closure of the mitral valve (LA end-systole). Images were analyzed using a commercially available software, cvi^42^ (version 13.5, Circle Cardiovascular Imaging Inc, Alberta, Calgary, Canada). The biplane area-length method [19] was applied to estimate the LA volumes by standard and LA-focused long-axis using the following formula:

$$LA volume=\frac{0.85\times {4}_{chamber}LA Area\times {2}_{chamber}LA Area }{{L}_{min}}$$

where L_min_ was the shorter long axis length of the LA either from the 2- or the 4-chamber view [19]. In the short-axis stack (used as reference) LA endocardial contours were manually traced and Simpson’s method of disks was applied for volumetric analysis. The most distal slice was defined as the slice showing LA cavity with no more than 50% of the circumference surrounded by LV myocardial tissue [10, 20]. The volume of the LA appendage was excluded from the LA cavity tracing, given its wide anatomical variability and its negligible clinical impact.

LAEF was calculated as (LAVmax − LAVmin)/LAVmax

To evaluate the clinical significance of using the three CMR image acquisition protocols, we categorized our patients in 4 groups based on previously published partition values: normal LA size (< 53 ml/m^2^), mildly dilated (53–62 ml/m^2^), moderately dilated (63–73 ml/m^2^), and severely dilated (> 73 ml/m^2^) [1].

LA strain analysis was performed using CMR feature-tracking (CMR-FT) on a dedicated software (Qstrain, version 2.1.12.2, Medis Medical Imaging Systems, Leiden, the Netherlands). LA endocardial borders were manually traced in 2- and 4-chamber views and the automated tracking algorithm was applied, with manual correction for minor misalignments. The three LA phasic functions (reservoir, conduit and booster pump) were analyzed.

### Statistical analysis

The normal distribution of the variables was assessed by Shapiro–Wilk normality test. Categorical variables are summarized as count and percentage. Normally distributed variables were summarized as mean ± standard deviation (SD), and non-normally distributed variables as median and interquartile range (IQR). Agreement between LA volumes and LAEF derived by both the standard long-axis and the LA-focused long-axis views with LA volumes and LAEF derived by short-axis stacks were analyzed using Pearson correlation and Bland–Altman plots, illustrating biases and 95% limits of agreement (LOA). LA volumes and LAEF obtained from the reference, the standard, and the LA-focused methods were compared using Kruskal–Wallis one-way analysis of variance. LA strain values obtained from the standard and the LA-focused methods were compared using Student T test.

Analyses were performed with SPSS (version 23.0, Statistical Package for the Social Sciences, International Business Machines, Inc., Armonk, New York, USA). A p-value < 0.05 was considered statistically significant.

#### Reproducibility analysis

Intra and inter-observer reproducibility analysis of LAVmax, LAVmin and LAEF measurements, obtained by reference and LA-focused imaging, was performed by two experienced operators (LT and SF, level 3 EACVI CMR certification) on 15 randomly chosen patients, by computing two-way mixed intraclass correlation coefficient (ICC) [21] for absolute agreement and Pearson correlation. An experienced operator reanalyzed the same data set weeks apart and blinded from the first measurements for intra-observer variability. A different experienced operator independently and blindly analyzed the same images for inter-observer variability.

## Results

We enrolled 118 patients. Ten patients were excluded: seven because of irregular heart rhythm at the time of the CMR study (either frequent ectopic beats or atrial fibrillation), and three due to the poor breath-hold performance during the study. The final study population consisted of 108 patients: 55 patients from Policlinico San Donato, and 53 patients from Istituto Auxologico Italiano. LA volumes could be evaluated in all patients (feasibility 100%). Out of 108 patients, 8 were excluded from LA strain analysis, due to inadequate semiautomatic tracking in either one or both the 2- and 4ch cine images.

Enrolled patients were predominantly men (71%), age of 55 ± 19 years.

Demographics, clinical indications to CMR, and CMR findings of the study cohort are summarized in Table 1.

**Table 1:** Clinical and CMR characteristics of the study population (n = 108)

Age (y)	55 ± 19
Body surface area (m^2^)	1.88 ± 0.21
Men (%)	71
Heart rate (bpm)	67 ± 13
LV end-diastolic volume (ml/m^2^)	82 ± 26
LV end-systolic volume(ml/m^2^)	33 ± 22
LV ejection fraction (%)	62 ± 12
LV mass (g/m^2^)	68 ± 19
RV end-diastolic volume (ml/m^2^)	75 ± 20
RV end-systolic volume (ml/m^2^)	27 ± 11
RV ejection fraction (%)	64 ± 8
Clinical indication to cardiac magnetic resonance (n, %)	
Ischaemic heart disease/myocardial viability	32 (29.6)
Assessment of cardiomyopathy phenotype	58 (53.7)
Assessment of causation of heart failure	16 (14.8)
Valvular heart disease	2 (1.9)

Data are expressed as mean ± standard deviations or as number and percentage

*LV* left ventricular, *RV* right ventricular

### LAVmax

The reference volumetric method yielded a LAVmax of 82 ml (IQR 67–105 ml) and a LAVmax i of 42 (IQR 36–54 ml/m^2^). LAVmax and LAV max-i derived from the standard cine images were 72 ml (IQR 55–93 ml) and 38 ml/m^2^ (IQR 28–48 ml/m^2^), respectively. LAVmax and LAVmax-i derived from LA-focused cine images were 83 ml (IQR 66–103 ml) and 43 ml/m^2^ (IQR 37–55 ml/m^2^), respectively.

The LAVmax obtained from the standard long-axis sequences were significantly smaller than the LAVmax obtained from both the reference (p = 0.007) and the LA-focused (p = 0.007) cine images. Conversely, the LAVmax obtained from the LA-focused images were similar to those obtained using the reference method (p = 1). Table 2.

**Table 2:** Comparison of left atrial volumes and emptying fraction from reference, standard, and focused-left-atrial cine images

	Short-axis cine images (Reference)	Standard Long axis cine images	LA-focused Long axis cine images	p-value
LAVmax (ml)	82 (67–105)	72 (55–93)* ^§^	83 (66–103)	0.002
LAVmin (ml)	38 (30–55)	29 (21–48)* ^§^	37 (27–53)	0.001
LAVmax-i (ml/m^2^)	42 (36–54)	38 (28–48)* ^§^	43 (37–55)	0.001
LAVmin-i (ml/m^2^)	20 (16–30)	16 (12–24)* ^§^	19 (15–28)	< 0.001
LAEF (%)	52 (45–58)	56 (48–64)*	55 (45–60)	0.008

Data are expressed as median (interquartile range). Kruskal–Wallis one-way analysis of variance (Dunn’s post hoc test). *p < 0.05 vs. Reference; ^§^p < 0.05 vs. LA-focused long axis

*LAEF* left atrial emptying fraction, *LAVmax* maximum left atrial volume, *LAVmin* minimum left atrial volume

Although a good correlation was noted between the standard and the reference methods (r^2^ = 0.86), Bland–Altman analysis revealed underestimation of LAVmax by the standard method (LAVmax bias: − 13 ml; 95% LOA: + 11 ml, − 37 ml; LAVmax-i bias: − 7 ml/m^2^; 95% LOA: + 7 ml/m^2^, − 21 ml/m^2^).

**Fig. 3:**
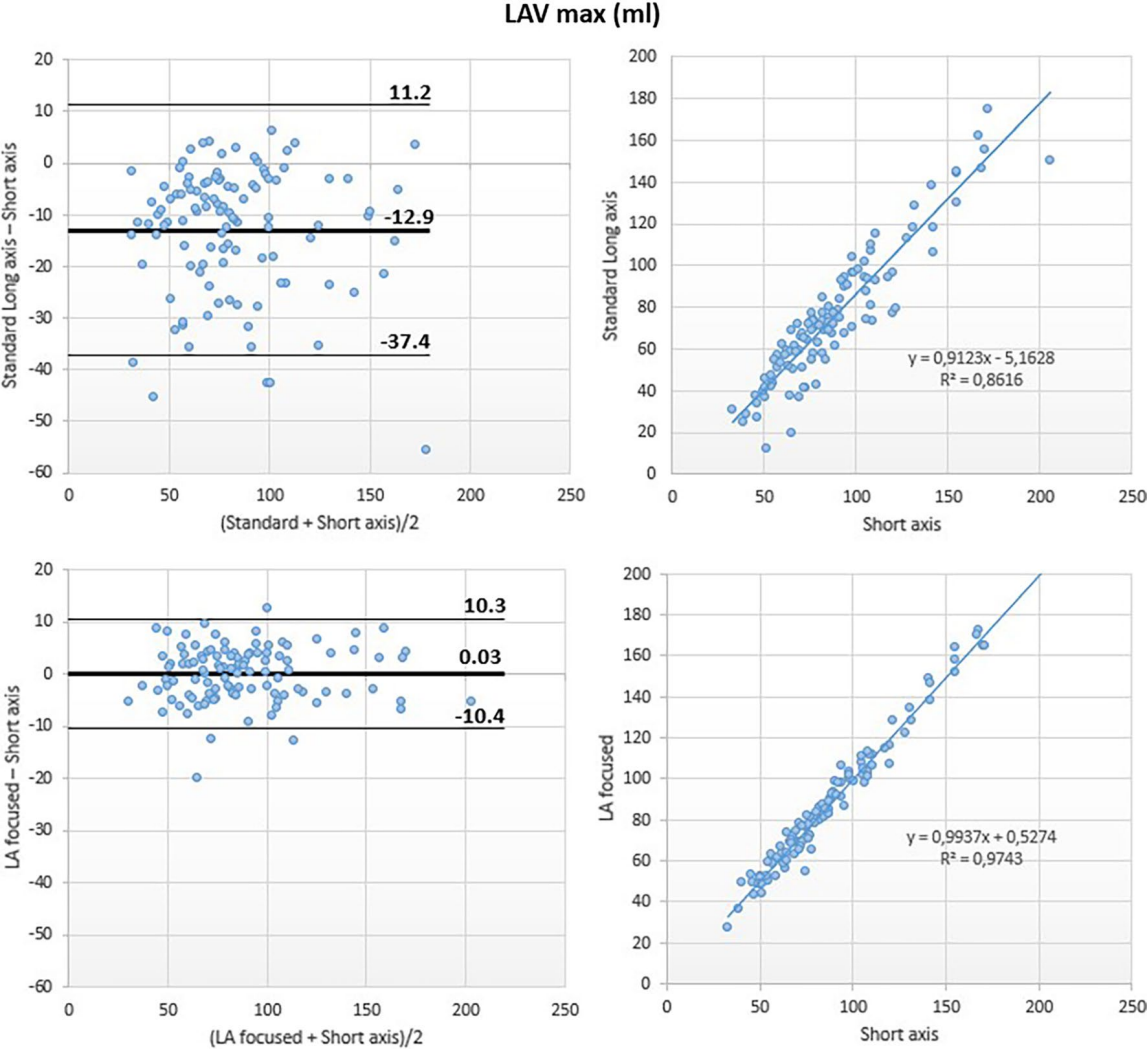
Left atrial maximum volume (LAVmax): comparison between methods. Comparison of LAVmax obtained using the biplane area-length method applied to both the standard (top panels) and the LA-focused (bottom panels) cine images, with LAVmax derived by the short-axis segmentation used as reference

Conversely, LA-focused and reference methods showed excellent correlation for LAVmax (r^2^ = 0.97) with negligible bias at Bland Altman analysis (LAVmax bias: 0 ml; 95% LOA: + 10 ml, − 10 ml; LAVmax i bias: 0 ml/m^2^; 95% LOA: + 5 ml/m^2^, − 6 ml/m^2^) (Figs. 3 and 7).

**Fig. 4:**
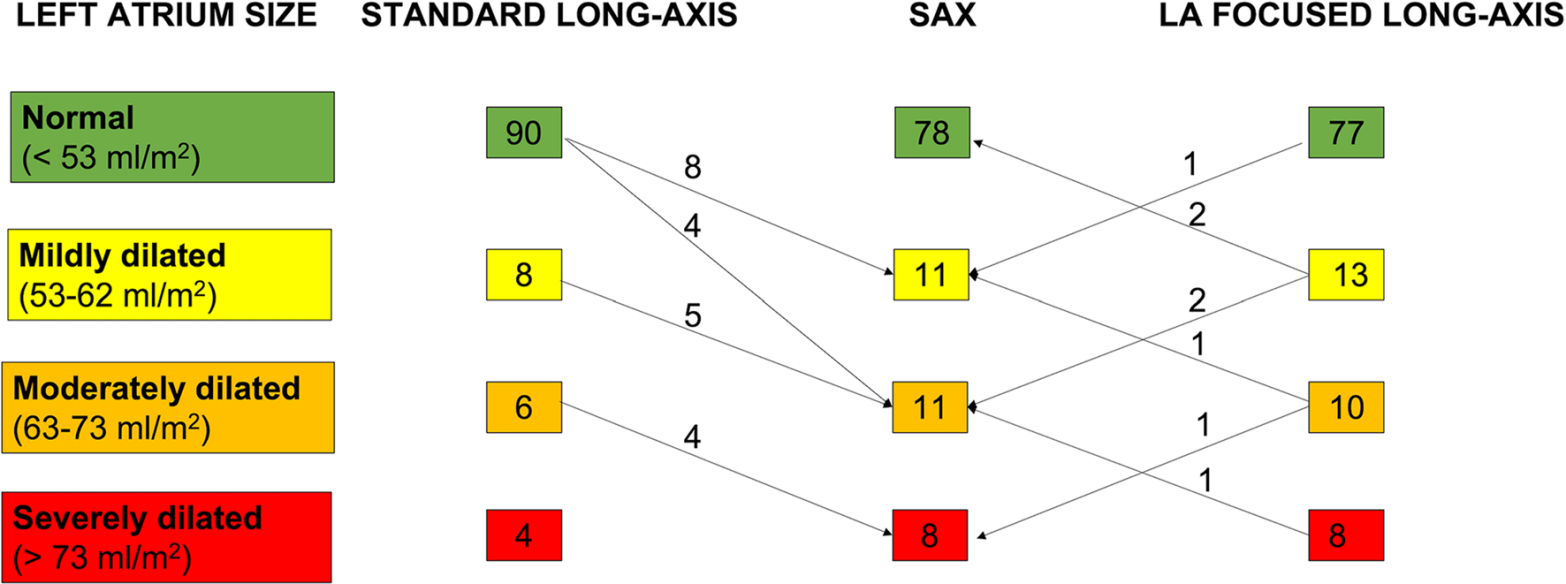
Distribution of the patients in LA size categories according to the method used for LAVmax quantification. The method using a short-axis stack of cine images covering the entire LA, from the atrio-ventricular ring to the base, was considered as reference. Arrows with superimposed numbers show the patients who change category by using either the standard or the LA-focused 4- and 2-chamber cine images

The extent of the underestimation of LAVmax-i obtained with the standard method determined a change of the category of LA dilation severity in 21 patients (19.4%). Among them, 9 patients who were classified as normal or mildly dilated with the standard method of LA volume measurements were found to have moderately dilated LA using the reference method, and 4 patients with moderately dilated LA according to the standard method were found to have severely dilated LA using the reference method. Compared to the standard method, by using the reference method to calculate the LA volumes moves 17 patients in one more severe degree and four patients in 2 more severe degrees of LA dilation. Conversely, only four patients had their LA dilation severity underestimated of only 1 degree using the LA-focused vs the reference method, whereas LA dilation severity was overestimated by 1 degree in four patients (Fig. 4).

### LAVmin

The reference volumetric method yielded a LAVmin of 38 ml (IQR 30–55 ml) and a LAVmin-i of 20 ml/m^2^ (IQR 16–30 ml/m^2^). The LAVmin and LAVmin-i derived from standard long-axis cine images were 29 ml (IQR 21–48 ml) and 16 ml/m^2^ (IQR 12–24 ml/m^2^), respectively. The LAVmin and LAVmin i derived from LA-focused long-axis cine images were 37 ml (IQR 27–53 ml) and 19 ml/m^2^ (IQR 15–28 ml/m^2^), respectively.

The LAVmin obtained from the standard long-axis sequences were significantly smaller than the LAVmin obtained from both the reference (p < 0.001) and the LA-focused (p = 0.01) cine images. Conversely, the LAVmin obtained from the LA-focused images were similar to those obtained from the reference method (p = 0.46) (Table 2).

Although a good correlation was noted between the LAVmin obtained using the standard and the reference methods (r^2^ = 0.87), Bland–Altman analysis revealed a large underestimation of LAVmin by the reference method (LAVmin bias: − 10 ml; 95% LOA: + 9 ml, − 28 ml; LAVmin-i bias: − 5 ml/m^2^; 95% LOA: + 5 ml/m^2^, − 16 ml/m^2^).

**Fig. 5:**
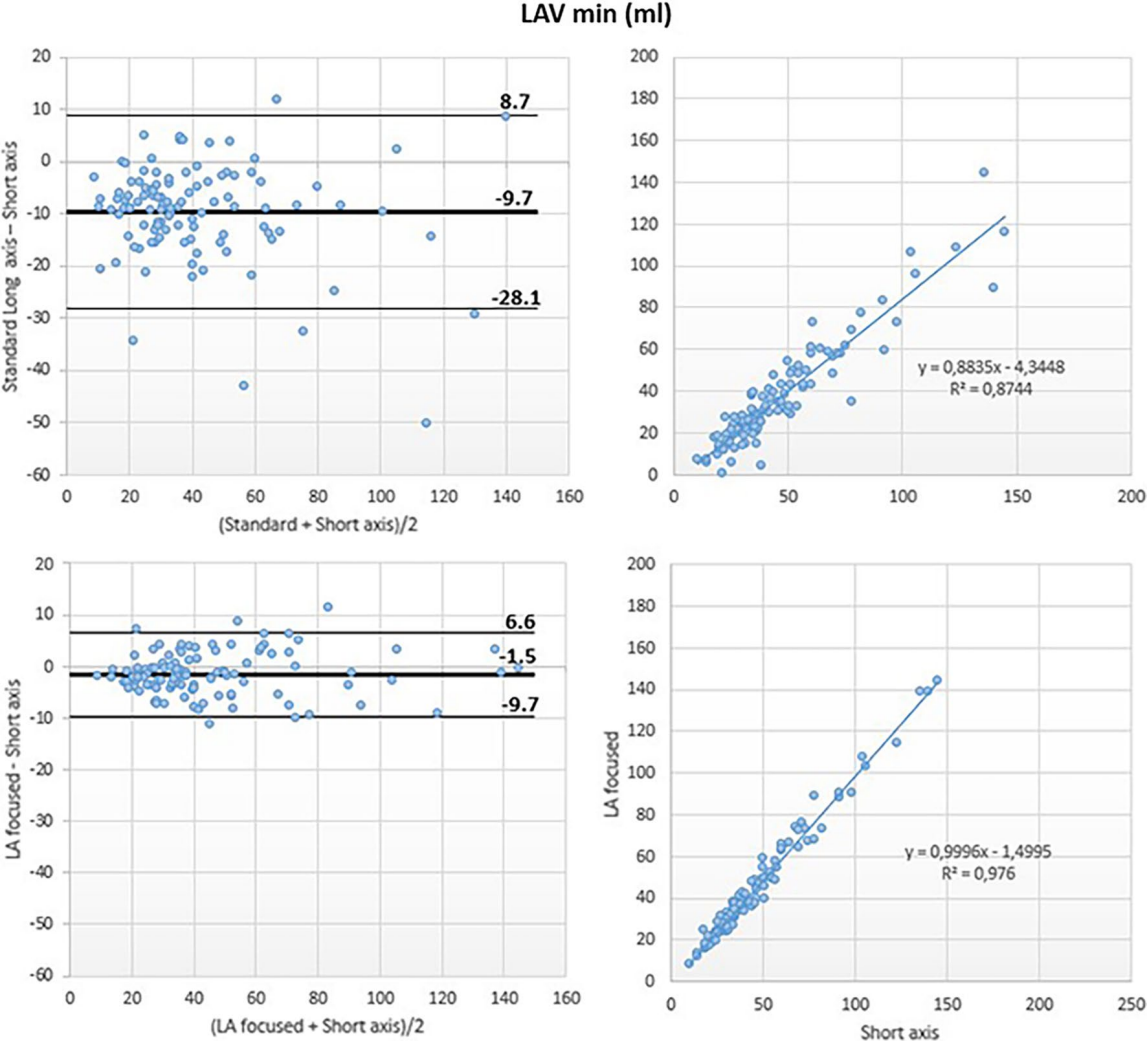
Left atrial minimum volume (LAVmin): comparison between methods. Comparison of LAVmin obtained using the biplane area-length method applied to both the standard (top panels) and the LA-focused (bottom panels) cine images, with LAVmin derived by the short-axis segmentation used as reference

Conversely, the LA-focused and the reference methods showed excellent correlation for LAVmin (r^2^ = 0.98) with negligible bias at Bland Altman analysis (LAVmin bias: − 2 ml, 95% LOA: + 7 ml, − 10 ml; LAVmin i bias: − 1 ml/m^2^, 95% LOA: + 3 ml/m^2^, − 5 ml/m^2^) (Figs. 5 and 8).

### LAEF

The reference volumetric method yielded a LAEF of 52% (IQR 45–58%). LAEF derived from the standard and the LA-focused long-axis cine images were 56% (IQR 48–64%) and 55% (IQR 45–60%), respectively.

The LAEF obtained using the standard long-axis sequences was significantly higher than the LAEF obtained from the reference method (p = 0.006). Conversely, the LAEF obtained from LA-focused images was similar to those obtained using the reference method (p = 0.3) (Table 2).

A moderate correlation was noted between the LAEF obtained using the standard and the reference methods (r^2^ = 0.58) and Bland–Altman analysis revealed overestimation of LAEF by the reference method (bias: 5%; 95% LOA: + 23%, − 14%).

**Fig. 6:**
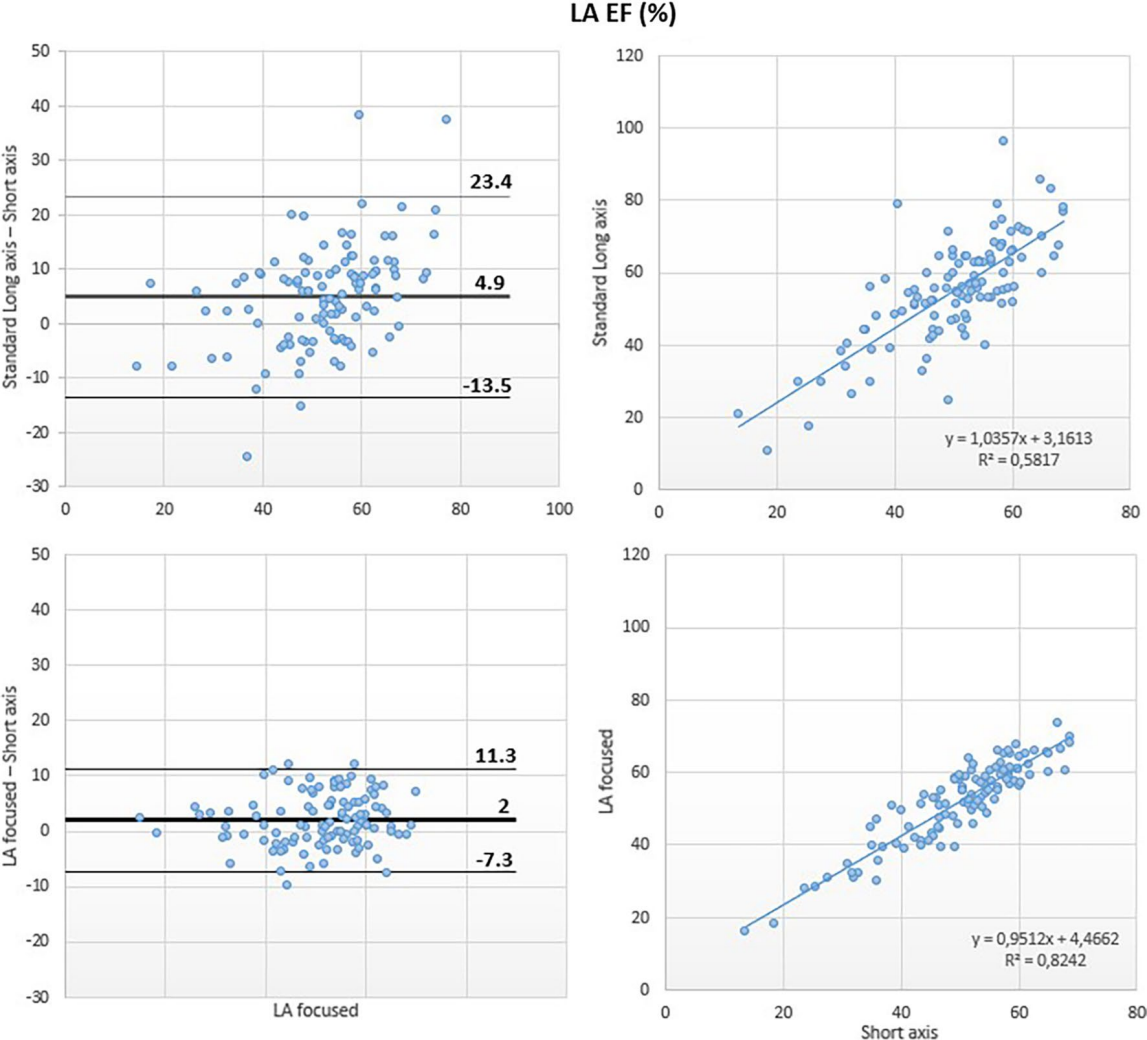
Left atrial emptying fraction: comparison between methods. Comparison of left atrial emptying fraction (LAEF) obtained using the biplane area-length method applied to both the standard (top panels) and the LA-focused (bottom panels) cine images, with LAEF derived by the short-axis segmentation that were taken as reference

Conversely, the LA-focused and the reference methods showed good correlation for LAEF (r^2^ = 0.82) with small bias at Bland Altman analysis (bias: 2%, 95% LOA: + 11, − 7%) (Fig. 6).

**Fig. 7:**
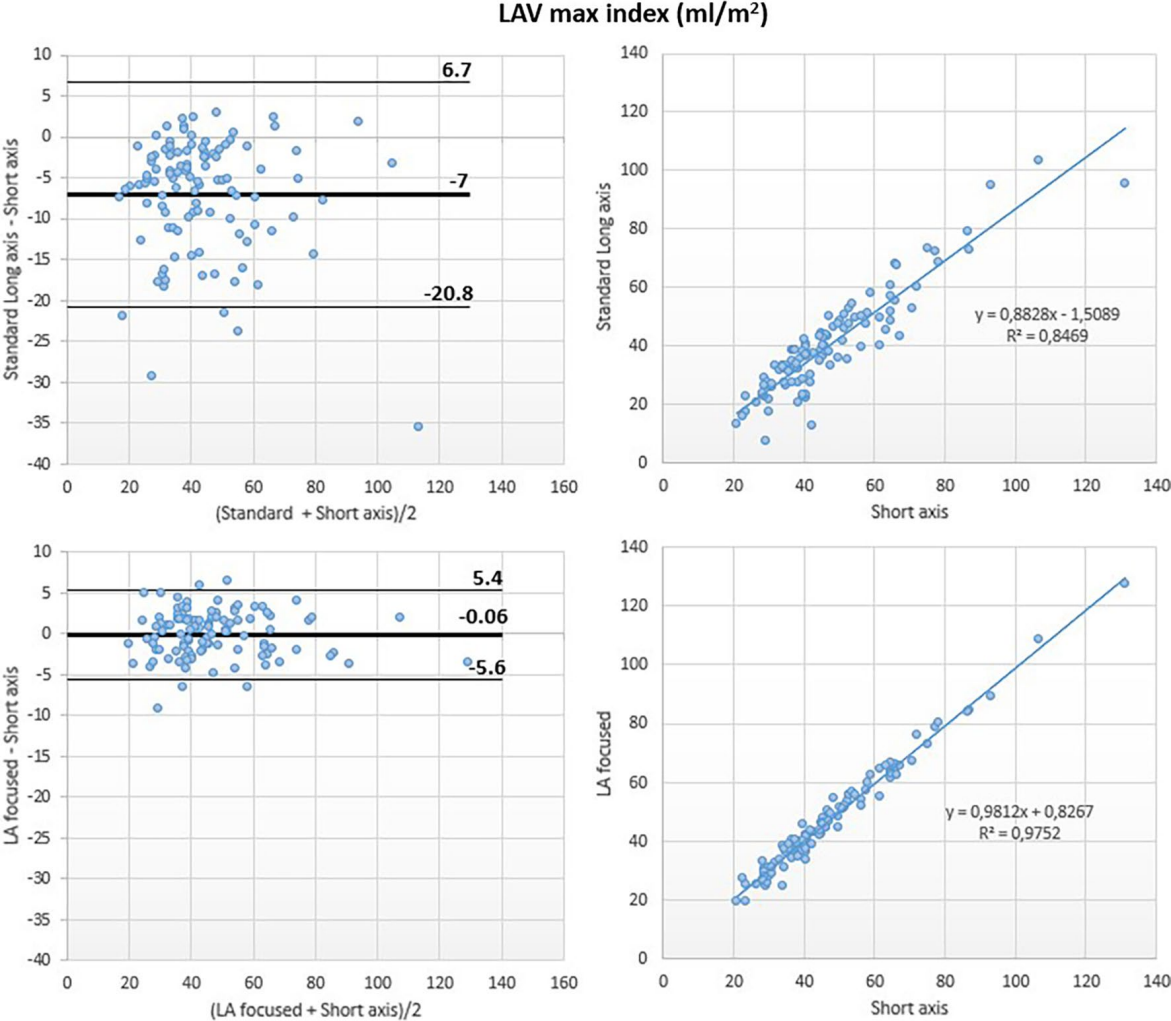
Left atrial maximum volume index (LAVmax-i): comparison between methods. Comparison of LAVmax-i obtained using the biplane area-length method applied to both the standard (top panels) and the LA-focused (bottom panels) cine images, with LAVmax-i derived by the short-axis segmentation used as reference

**Fig. 8:**
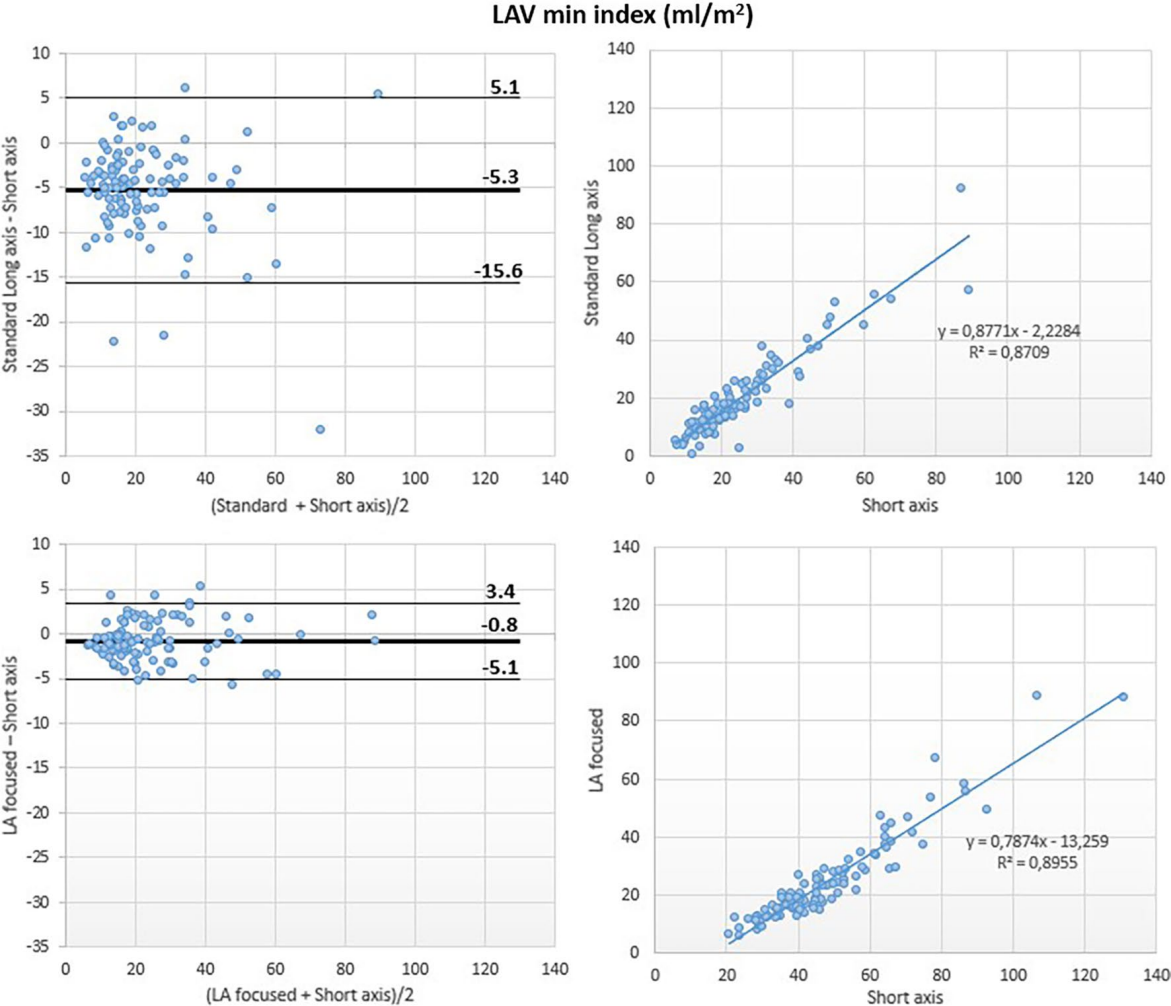
Left atrial minimum volume index (LAVmin-i): comparison between methods. Comparison of LAVmin-I obtained using the biplane area-length method applied to both the standard (top panels) and the LA-focused (bottom panels) cine images, with LAVmin-i derived by the short-axis segmentation used as reference

### Reproducibility of LA size and function by LA-focused long-axis views

LAVmax, LAVmin and LAEF measured using the LA-focused long-axis views showed excellent intra- and inter-observer reproducibility, with ICC ranging from 0.97 to 0.99 (Table 3).

**Table 3:** Intra- and inter-observer reproducibility for left atrial volumes and emptying fraction from LA-focused cine images

	ICC Intra-Observer (CI 95%)	ICC Inter-Observer (CI 95%)	Pearson (r) Intra-Observer	Pearson (r) Inter-Observer
LAVmax (ml)	0.99 (0.98–0.99)	0.99 (0.97–0.99)	0.99	0.98
LAVmin (ml)	0.99 (0.98–0.99)	0.99 (0.98–0.99)	0.99	0.98
LAEF (%)	0.97 (0.91–0.98)	0.97 (0.92–0.99)	0.93	0.94

*CI* confidence interval, *LAEF* left atrial emptying fraction, *ICC* intraclass correlation coefficient, *LAEF* left atrial emptying fraction, *LAVmax* maximum left atrial volume, *LAVmin* minimum left atrial volume

LAVmax, LAVmin and LAEF measured using the reference short-axis stack showed good to excellent intra- and inter-observer reproducibility, with ICC ranging from 0.88 to 0.99 (Additional file 1: Table S1).

### LA strain analysis

At CMR-FT analysis, all three LA strain values (reservoir strain (εs): bias 7%, LOA = 25, − 11%; conduit strain (εe): bias 4%, LOA = 15, − 8%; booster pump strain (εa): bias 3%, LOA = 14, − 8%; all p < 0.001) were significantly higher in standard vs. LA-focused images. LA strain values showed moderate correlation between the two methods (Table 4).

**Table 4:** CMR feature tracking left atrial strain analysis on LA-focused vs. standard cine images

	Standard	LA-focused	Bias (LOA)	Pearson (r)
Reservoir (εs) (%)	31 ± 12	25 ± 8	7 (25, − 11)	0.63
Conduit (εe) (%)	16 ± 9	12 ± 6	4 (15, − 8)	0.76
Booster pump (εa) (%)	15 ± 6	12 ± 5	3 (14, − 8)	0.55

### CMR scan acquisition delay and post-processing time related to LA volumes quantification

The scan delay due to the acquisition of the LA-focused long-axis cine images was significantly shorter than the scan delay due to short-axis LA acquisition, used as reference method, (72 s, IQR 60–120 s vs. 270 s, IQR 228–300; p < 0.001).

The post-processing time of LA-focused cine images was significantly shorter than that of reference short-axis LA stack (28 s, IQR 25–29 s vs. 103 s, IQR 98–129 s; p = 0.008) and similar to that of standard long-axis cine images (26 s, IQR 24–28 s; p = 0.4).

## Discussion

To our knowledge, this is the first study proposing and testing the accuracy and reproducibility of CMR LA dedicated long-axis cine views to measure LA volumes and LAEF, and to assess the impact on LA strain values of using the LA focused images.

Our results can be summarized as follows: (i) LA volumes and LAEF obtained by LA-focused imaging did not significantly differ from the respective values obtained by the reference method in patients undergoing clinically indicated CMR; (ii) LA volumes were significantly larger, and LAEF was significantly lower, when they were obtained from LA-focused long-axis cine images compared to standard long-axis views; (iii) LA strain values obtained by LA-focused imaging are significantly lower than those produced by standard images; (iv) LA volumes and LAEF obtained by LA-focused imaging showed excellent intra- and inter-observer reproducibility and (v) the scan delay and the post-processing time due to LA-focused images acquisition and analysis were significantly lower than those required by the short-axis stack used as a reference.

LA remodeling largely depends on LV diastolic performance over time [3, 4], represents an early feature of several cardiovascular diseases [4, 22] and heralds adverse cardiovascular outcomes [2, 11, 12].

Among the various LA imaging parameters, LAVmax has been the most extensively assessed biomarker [1–5]. More recently, there has been increasing interest for LAVmin, which has shown a greater prognostic value than LAVmax [23–25] and may reflect the ‘contractile’ function of the LA more precisely [26, 27]. LAEF is another relevant parameter, since it showed the best correlation with E/Eʹ values in a population of patients with diastolic dysfunction assessed by echocardiography [28]. LA strain is also emerging as a prognosticator of adverse cardiovascular events in multiple disease settings [29, 30] and in the general population [31].

At present, CMR is considered the gold standard for morpho-functional assessment of cardiac chambers [7]. However, current CMR guidelines do not provide specific recommendations on how to measure LA volumes, LAEF and strain.

LA volumes are usually calculated by using the biplane area-length algorithm in standard (i.e. LV focused) long-axis cine images [1, 2, 5, 12–14, 32, 33]. However, LV and LA long-axis are not parallel [10]. Thus, if LA volumes are measured in standard long-axis images, which optimize the orientation for the LV, there is a likelihood of foreshortening the LA images, entailing an underestimation of LA volumes [9, 34–36] and an overestimation of LA strain [17]. These methodological issues are critical to diagnose LA volumes dilation and/or dysfunction using tomographic imaging techniques [9, 10, 16]. The use of the standard method was associated to the underestimation of the severity of LA dilation in 21% of our patients (the underestimation was of two degrees of severity in 4%), that may be associated to a significant underestimation of both their mortality and morbidity [1, 5].

LA volumetric analysis by CMR has been considered as the reference standard in several investigations, by performing a short-axis stack of cine images covering the LA, and then calculating LA volumes by tracing LA cavity at end-systole and end-diastole [9, 11, 14]. Unfortunately, this approach is time-consuming and is usually limited to research purposes. Accordingly, the best CMR methodological approach for measuring LA volumes and LAEF in daily practice remains to be established.

In the present study, we propose a new method consisting of applying the biplane area-length method to dedicated long-axis cine views, focused on the LA and intercepting its true long-axis. In comparison to the biplane area-length method applied to standard long-axis views, we demonstrated that this approach, which avoids LA foreshortening, provides a more accurate estimation of LA volumes, with values that were significantly larger than those obtained using the standard (i.e. LV-focused) cine images. Moreover, LAEF assessed by standard long-axis sequences showed only a moderate correlation with the values obtained from the reference method, resulting in significant overestimation of LA function. Conversely, LAEF values derived from LA-focused images and the reference method were similar.

Iwataki et al. [16] were the first to demonstrate that conventional apical long-axis views by 2D echocardiography were less accurate than LA dedicated 4- and 2-chamber views to estimate LA volumes measured by 3D echocardiography. Badano et al. [10] confirmed the underestimation of LA volumes measured by 3D echocardiography by the biplane area-length method applied to conventional apical 4- and 2-chamber views obtained by 2D echocardiography. Accordingly, current guidelines for the assessment of cardiac chambers by 2D echocardiography recommend the use of dedicated, LA-focused apical 4- and 2-chamber views to assess LA volumes [15].

The evaluation of LA size and function by CMR has gained increasing attention in the last years. The potential role of area-length biplane algorithm as an alternative and faster method to Simpson’s for the volumetric analysis of the LA has been repeatedly explored, providing controversial results.

Sievers et al. [19] suggested that LA volumes and LAEF calculated through the biplane area-length method from standard long-axis images are accurate in healthy subjects and patients with atrial fibrillation. Consistently, the authors found marginally larger LAVmax (difference of 1 ± 1 ml) obtained by the biplane area-length method than those calculated by the Simpson’s method in short-axis, whereas LAVmin and LAEF were similar between the two methods. These findings were derived from a single-center population including 33 individuals. Atrial volumes were obtained through prospective gating and bSSFP, which do not represent the current CMR standard of practice [8].

Nacif et al. [36] found that, in 88 patients with a history of atrial fibrillation, the LA volumes and LAEF calculated using the biplane area-length method did not significantly differ from those obtained by the Simpson’s method applied to short-axis derived by retrospective gating and bSSFP sequences.

However, Nanni et al. [34] subsequently reported that LAVmin, although not LAVmax, was significantly underestimated by the standard biplane area-length, entailing a significant overestimation of LAEF in comparison to the short axis Simpson’ method.

Wandelt et al. [9] demonstrated a relevant underestimation of both LA volumes and confirmed an overestimation of the LAEF through the biplane area-length-algorithm applied to standard long-axis cine images vs. the Simpson’s method applied to a stack of slices covering the LA in transversal orientation as a reference (80 vs. 99 ml, 37 vs. 53 ml and 55 vs. 48% for LAVmax, LAVmin and LAEF, respectively).

Our findings are in line with the results of the latter two studies, showing an underestimation of LA volumes and an overestimation of LAEF obtained through the biplane-area length algorithm applied on standard cine images in comparison to the reference method.

As regards LA strain, CMR-FT is a feasible and reproducible semi-automated post-processing method using bSSFP to quantitatively assess LA phasic functions (reservoir, conduit and booster pump) [37]. Nabeshima et al. [17] recently reported that the foreshortening of the LA cavity in 2D echo apical views potentially overestimates reservoir strain. CMR-FT-derived LA strain values are usually calculated using standard long-axis cine images [38]. Our study is the first to apply CMR-FT analysis to both standard and LA-focused long-axis views. We showed that all three LA strain components obtained by LA-focused images are significantly lower than those obtained using the standard 2- and 4-chamber cine images. The difference observed may be partly explained by the fact that strain values depend on the geometry of the chamber, which varies according to the acquisition plane. Moreover, LA-focused images better include LA posterior wall, where pulmonary veins convey and atrial deformation is probably blunted.

To try to maintain the convenient workflow and the time-saving approach provided with the biplane area-length algorithm, and take into account the different anatomical orientation of the LA compared to the LV, we propose to adopt for CMR an approach similar to the one recommended for 2D echocardiography. The acquisition of dedicated, LA focused (i.e. optimized for the LA length) 4- and 2 chamber views required a very short extra scan time, was significantly faster than the short-axis method, and provided similar results to the short axis reference method with excellent intra- and inter-observer reproducibility. The latter feature is essential for the potential clinical implementation since imaging speed is a major issue of CMR [39].

### Limitations

This is a pilot and methodological investigation. Thus, we do not provide normal reference values for the proposed acquisition protocol of the LA, which will eventually be the subject of a future investigation.

We did not use an independent cardiac imaging modality as reference for the accuracy of LA volumes. In fact, cardiac computed tomography entails radiation exposure and may be unethical without a specific clinical indication. Nevertheless, we validated LA-focused long-axis cine images with LA volumes obtained by a CMR short-axis cine stack, which is considered as a reference standard.

The present study is purely methodological, as it evaluates the accuracy of LA volumes obtained from the LA-focused cine images compared to standard long-axis ones. The clinical impact of this novel approach is beyond the aims of the study. According to our results, and the emerging literature about the prognostic role of LA size and function assessed by CMR, the clinical impact of LA-focused cine sequences warrants further investigation.

Finally, the biplane area-length algorithm is based on the geometric assumption of an ellipsoid chamber. Although we have enrolled a relatively large number of patients with a wide range of LA volumes, future studies are needed to confirm the robustness of our results in the various geometries of the LA.

## Conclusions

CMR assessment of the LA using LA-focused long-axis cine imaging is quick, feasible and provides more accurate LA volumes and LAEF than standard long-axis cine imaging. LA strain values obtained by LA-focused imaging are significantly lower than those produced by standard images. Considering the diagnostic and prognostic value of the accurate assessment of LA size in many cardiac conditions, and the limited additional scan time needed to acquire dedicated LA-focused images, this method should enter the clinical routine of CMR studies.

## Supplementary Information

**Additional file 1:  Table S1.** Intra- and inter-observer reproducibility for left atrial volumes and emptying fraction from short axis manual segmentation (reference). **Table S2.** Bland Altman and correlation analysis between standard and left atrial-focused long axis images for left atrial volumes, emptying fraction and diameters. **Table S3.** Comparison of left atrial long-axis diameters between standard and LA-focused images.

## Data Availability

The datasets used and/or analyzed during the current study are available from the corresponding author on reasonable request.

## Abbreviations

2D: Two-dimensional
3D: Three-dimensional
bSSFP: Balanced steady state free precession
CMR: Cardiovascular magnetic resonance
εa: Booster pump strain
εe: Conduit strain
εs: Reservoir strain
FT: Feature tracking
ICC: Intraclass correlation coefficient
IQR: Interquartile range
LA: Left atrium/left atrial
LAEF: Left atrial emptying fraction
LAVmax: Maximum left atrial volume
LAVmax-i: Maximum left atrial indexed to body surface area
LAVmin: Minimum left atrial volume
LAVmin-i: Minimum left atrial volume indexed to body surface area
LOA: Limits of agreement
LV: Left ventricle/left ventricular
LVEF: Left ventricular ejection fraction
LVM: Left ventricular mass
RV: Right ventricle/right ventricular
RVEF: Right ventricular ejection fraction

## References

[1] Khan MA, Yang EY, Zhan Y, Judd RM, Chan W, Nabi F (2019). Association of left atrial volume index and all-cause mortality in patients referred for routine cardiovascular magnetic resonance: a multicenter study. J Cardiovasc Magn Reson.

[2] Gulati A, Ismail TF, Jabbour A, Ismail NA, Morarji K, Ali A, Raza S (2013). Clinical utility and prognostic value of left atrial volume assessment by cardiovascular magnetic resonance in non-ischaemic dilated cardiomyopathy. Eur J Heart Fail.

[3] Thomas L, Marwick TH, Popescu BA, Donal E, Badano LP (2019). Left atrial structure and function, and left ventricular diastolic dysfunction: JACC state-of-the-art review. J Am Coll Cardiol.

[4] Thomas L, Muraru D, Popescu BA, Sitges M, Rosca M, Pedrizzetti G (2020). Evaluation of left atrial size and function: relevance for clinical practice. J Am Soc Echocardiogr.

[5] Raisi-Estabragh Z, McCracken C, Condurache D, Aung N, Vargas JD, Naderi H et al. Left atrial structure and function are associated with cardiovascular outcomes independent of left ventricular measures: a UK Biobank CMR study. Eur Heart J Cardiovasc Imaging. 2021;jeab266. 10.1093/ehjci/jeab266 PMC9365306 34907415

[6] Garg P, Gosling R, Swoboda P, Jones R, Rothman A, Wild JM (2022). Cardiac magnetic resonance identifies raised left ventricular filling pressure: prognostic implications. Eur Heart J.

[7] Petersen SE, Khanji MY, Plein S, Lancellotti P, Bucciarelli-Ducci C (2019). European Association of Cardiovascular Imaging expert consensus paper: a comprehensive review of cardiovascular magnetic resonance normal values of cardiac chamber size and aortic root in adults and recommendations for grading severity. Eur Heart J Cardiovasc Imaging.

[8] Kramer CM, Barkhausen J, Bucciarelli-Ducci C, Flamm SD, Kim RJ, Nagel E (2020). Standardized cardiovascular magnetic resonance imaging (CMR) protocols: 2020 update. J Cardiovasc Magn Reson.

[9] Wandelt LK, Kowallick JT, Schuster A, Wachter R, Stümpfig T, Unterberg-Buchwald C (2017). Quantification of left atrial volume and phasic function using cardiovascular magnetic resonance imaging-comparison of biplane area-length method and Simpson's method. Int J Cardiovasc Imaging.

[10] Badano LP, Miglioranza MH, Mihăilă S, Peluso D, Xhaxho J, Marra MP (2016). Left atrial volumes and function by three-dimensional echocardiography: reference values, accuracy, reproducibility, and comparison with two-dimensional echocardiographic measurements. Circ Cardiovasc Imaging.

[11] Lønborg JT, Engstrøm T, Møller JE, Ahtarovski KA, Kelbæk H, Holmvang L (2013). Left atrial volume and function in patients following ST elevation myocardial infarction and the association with clinical outcome: a cardiovascular magnetic resonance study. Eur Heart J Cardiovasc Imaging.

[12] Patel RK, Jardine AG, Mark PB, Cunningham AF, Steedman T, Powell JR (2010). Association of left atrial volume with mortality among ESRD patients with left ventricular hypertrophy referred for kidney transplantation. Am J Kidney Dis.

[13] Hudsmith LE, Petersen SE, Francis JM, Robson MD, Neubauer S (2005). Normal human left and right ventricular and left atrial dimensions using steady state free precession magnetic resonance imaging. J Cardiovasc Magn Reson.

[14] Kawel-Boehm N, Maceira A, Valsangiacomo-Buechel ER, Vogel-Claussen J, Turkbey EB, Williams R (2015). Normal values for cardiovascular magnetic resonance in adults and children. J Cardiovasc Magn Reson.

[15] Lang RM, Badano LP, Mor-Avi V, Afilalo J, Armstrong A, Ernande L (2015). Recommendations for cardiac chamber quantification by echocardiography in adults: an update from the American Society of Echocardiography and the European Association of Cardiovascular Imaging. J Am Soc Echocardiogr.

[16] Iwataki M, Takeuchi M, Otani K, Kuwaki H, Haruki N, Yoshitani H (2012). Measurement of left atrial volume from transthoracic three-dimensional echocardiographic datasets using the biplane Simpson’s technique. J Am Soc Echocardiogr.

[17] Nabeshima Y, Kitano T, Takeuchi M (2021). Reliability of left atrial strain reference values: a 3D echocardiographic study. PLoS ONE.

[18] Perez de Isla L, Feltes G, Moreno J, Martinez W, Saltijeral A, de Agustin JA (2014). Quantification of left atrial volumes using three-dimensional wall motion tracking echocardiographic technology: comparison with cardiac magnetic resonance. Eur Heart J Cardiovasc Imaging.

[19] Sievers B, Kirchberg S, Addo M, Bakan A, Brandts B, Trappe HJ (2004). Assessment of left atrial volumes in sinus rhythm and atrial fibrillation using the biplane area-length method and cardiovascular magnetic resonance imaging with TrueFISP. J Cardiovasc Magn Reson.

[20] Mor-Avi V, Yodwut C, Jenkins C, Kühl H, Nesser HJ, Marwick TH (2012). Real-time 3D echocardiographic quantification of left atrial volume: multicenter study for validation with CMR. JACC Cardiovasc Imaging.

[21] Koo TK, Li MY. A guideline of selecting and reporting intraclass correlation coefficients for reliability research. J Chiropr Med. 2016;15(2):155–63. doi: 10.1016/j.jcm.2016.02.012. Epub 2016 Mar 31. Erratum in: J Chiropr Med. 2017;16(4):346. 10.1016/j.jcm.2016.02.012 PMC4913118 27330520

[22] Bernardini A, Camporeale A, Pieroni M, Pieruzzi F, Figliozzi S, Lusardi P (2020). Atrial dysfunction assessed by cardiac magnetic resonance as an early marker of fabry cardiomyopathy. JACC Cardiovasc Imaging.

[23] Russo C, Jin Z, Homma S, Rundek T, Elkind MSV, Sacco RL, Di Tullio MR (2017). LA phasic volumes and reservoir function in the elderly by real-time 3D echocardiography: normal values, prognostic significance, and clinical correlates. JACC Cardiovasc Imaging.

[24] Wu VC, Takeuchi M, Kuwaki H, Iwataki M, Nagata Y, Otani K (2013). Prognostic value of LA volumes assessed by transthoracic 3D echocardiography: comparison with 2D echocardiography. JACC Cardiovasc Imaging.

[25] Matei LL, Ghilencea LN, Bejan GC, Stoica S, Dragoi-Galrinho R, Siliste C (2021). Minimum left atrial volume evaluated by 3D echocardiography predicts atrial fibrillation recurrences after a first radiofrequency catheter ablation for paroxysmal episodes. Maedica (Bucur).

[26] Abhayaratna WP, Seward JB, Appleton CP, Douglas PS, Oh JK, Tajik AJ (2006). Left atrial size: physiologic determinants and clinical applications. J Am Coll Cardiol United States.

[27] Aune E, Baekkevar M, Roislien J, Rodevand O, Otterstad JE (2009). Normal reference ranges for left and right atrial volume indexes and ejection fractions obtained with real-time three-dimensional echocardiography. Eur J Echocardiogr J Work Gr Echocardiogr Eur Soc Cardiol England.

[28] Murata M, Iwanaga S, Tamura Y, Kondo M, Kouyama K, Murata M (2008). A real-time three-dimensional echocardiographic quantitative analysis of left atrial function in left ventricular diastolic dysfunction. Am J Cardiol.

[29] Santos AB, Roca GQ, Claggett B, Sweitzer NK, Shah SJ, Anand IS (2016). Prognostic relevance of left atrial dysfunction in heart failure with preserved ejection fraction. Circ Heart Fail.

[30] Lelong B, Chabanne C, Fournet M, Galli E, Mabo P, Donal E (2015). Prognostic value of left atrial reservoir function in patients with severe aortic stenosis: a 2D speckle-tracking echocardiographic study. Eur Heart J Cardiovasc Imaging.

[31] Modin D, Biering-Sørensen SR, Møgelvang R, Alhakak AS, Jensen JS, Biering-Sørensen T (2019). Prognostic value of left atrial strain in predicting cardiovascular morbidity and mortality in the general population. Eur Heart J Cardiovasc Imaging.

[32] Maceira AM, Cosín-Sales J, Roughton M, Prasad SK, Pennell DJ (2010). Reference left atrial dimensions and volumes by steady state free precession cardiovascular magnetic resonance. J Cardiovasc Magn Reson.

[33] Kühl JT, Lønborg J, Fuchs A, Andersen MJ, Vejlstrup N, Kelbæk H (2012). Assessment of left atrial volume and function: a comparative study between echocardiography, magnetic resonance imaging and multi slice computed tomography. Int J Cardiovasc Imaging.

[34] Nanni S, Westenberg JJ, Bax JJ, Siebelink HM, de Roos A, Kroft LJ (2016). Biplane versus short-axis measures of the left atrium and ventricle in patients with systolic dysfunction assessed by magnetic resonance. Clin Imaging.

[35] Hudsmith LE, Cheng AS, Tyler DJ, Shirodaria C, Lee J, Petersen SE (2007). Assessment of left atrial volumes at 1.5 Tesla and 3 Tesla using FLASH and SSFP cine imaging. J Cardiovasc Magn Reson..

[36] Nacif MS, Barranhas AD, Türkbey E, Marchiori E, Kawel N, Mello RA (2013). Left atrial volume quantification using cardiac MRI in atrial fibrillation: comparison of the Simpson's method with biplane area-length, ellipse, and three-dimensional methods. Diagn Interv Radiol.

[37] Kowallick JT, Morton G, Lamata P, Jogiya R, Kutty S, Hasenfuß G (2015). Quantification of atrial dynamics using cardiovascular magnetic resonance: interstudy reproducibility. J Cardiovasc Magn Reson.

[38] Truong VT, Palmer C, Wolking S, Sheets B, Young M, Ngo TNM (2020). Normal left atrial strain and strain rate using cardiac magnetic resonance feature tracking in healthy volunteers. Eur Heart J Cardiovasc Imaging.

[39] Axel L, Otazo R (2016). Accelerated MRI for the assessment of cardiac function. Br J Radiol..

