# Supplementary material for: The use of dedicated long-axis views focused on the left atrium improves the accuracy of left atrial volumes and emptying fraction measured by cardiovascular magnetic resonance
Source: J Cardiovasc Magn Reson. 2023 Feb 16;25:10. doi: 10.1186/s12968-022-00905-w (PMC9933380; doi:10.1186/s12968-022-00905-w)
Supplement: Supplementary file 1 — Additional file 1: Table S1. Intra- and inter-observer reproducibility for left atrial volumes and emptying fraction from short axis manual segmentation (reference). Table S2. Bland Altman and correlation analysis between standard and left atrial-focused long axis images for left atrial volumes, emptying fraction and diameters. Table S3. Comparison of left atrial long-axis diameters between standard and LA-focused images. [file 12968_2022_905_MOESM1_ESM.docx]

**Table 1s. Intra- and inter-observer reproducibility for left atrial volumes and emptying fraction from *short axis* manual segmentation (*reference*)**

|  | **ICC Intra-Observer (CI 95%)** | **ICC Inter-Observer (CI 95%)** | **Pearson (r) Intra-Observer** | **Pearson (r) Inter-Observer** |
| --- | --- | --- | --- | --- |
| **LAVmax (ml)** | 0.98 (0.96-0.99) | 0.97 (0.95-0.99) | 0.96 | 0.96 |
| **LAVmin (ml)** | 0.98 (0.97-0.99) | 0.97 (0.94-0.98) | 0.97 | 0.95 |
| **LAEF (%)** | 0.97 (0.94-0.99) | 0.88 (0.77-0.95) | 0.94 | 0.82 |

*Abbreviations: CI, confidence interval; EF, emptying fraction; ICC, intraclass correlation coefficient LAEF, left atrial emptying fraction; LAVmax, maximum left atrial volume; LAVmin, minimum left atrial volume.*

**Table 2s. Bland Altman and correlation analysis between *standard* and *left atrial-focused* long axis images for left atrial volumes, emptying fraction and diameters**

|  | **Bias (upper and lower LOA)** | **Pearson (r)** |
| --- | --- | --- |
| **LAVmax (ml)** | -13 (11, -37) | 0.93 |
| **LAVmin (ml)** | -8 (9, -26) | 0.94 |
| **LAVmax-i (ml/m^2^)** | -7 (6, -20) | 0.92 |
| **LAVmini (ml/m^2^)** | -4 (6, -14) | 0.94 |
| **LAEF (%)** | 3 (20, -14) | 0.81 |
| **LA max diameter (mm)** | -9 (5, -23) | 0.56 |
| **LA min diameter (mm)** | -8 (6, -22) | 0.66 |

*LAVmax-i, maximum left atrial volume indexed to body surface area; LAVmin-i, minimum left atrial volume indexed to body surface area.*

**Table 3s. Comparison of left atrial long-axis diameters between *standard* and *LA-focused images***

|  | **Standard LV Long Axis cine images** | **LA-focused Long Axis cine images** | **p-value** |
| --- | --- | --- | --- |
| **LA max diameter (mm)** | 50 (47-56) | 60 (55-64) | < 0.001 |
| **LA min diameter (mm)** | 38 (33-43) | 46 (42-51) | < 0.001 |
